# Enrofloxacin—The Ruthless Killer of Eukaryotic Cells or the Last Hope in the Fight against Bacterial Infections?

**DOI:** 10.3390/ijms23073648

**Published:** 2022-03-26

**Authors:** Łukasz Grabowski, Lidia Gaffke, Karolina Pierzynowska, Zuzanna Cyske, Marta Choszcz, Grzegorz Węgrzyn, Alicja Węgrzyn

**Affiliations:** 1 Laboratory of Phage Therapy, Institute of Biochemistry and Biophysics, Polish Academy of Sciences, Kładki 24, 80-822 Gdansk, Poland; l.grabowski@ibb.waw.pl; 2 Department of Molecular Biology, Faculty of Biology, University of Gdansk, Wita Stwosza 59, 80-308 Gdansk, Poland; lidia.gaffke@ug.edu.pl (L.G.); karolina.pierzynowska@ug.edu.pl (K.P.); zuzanna.cyske@phdstud.ug.edu.pl (Z.C.); marta.choszcz@gmail.com (M.C.); grzegorz.wegrzyn@biol.ug.edu.pl (G.W.)

**Keywords:** enrofloxacin, antibiotic, safety, efficacy, adverse effects, toxicity

## Abstract

Enrofloxacin is a compound that originates from a group of fluoroquinolones that is widely used in veterinary medicine as an antibacterial agent (this antibiotic is not approved for use as a drug in humans). It reveals strong antibiotic activity against both Gram-positive and Gram-negative bacteria, mainly due to the inhibition of bacterial gyrase and topoisomerase IV enzymatic actions. The high efficacy of this molecule has been demonstrated in the treatment of various animals on farms and other locations. However, the use of enrofloxacin causes severe adverse effects, including skeletal, reproductive, immune, and digestive disorders. In this review article, we present in detail and discuss the advantageous and disadvantageous properties of enrofloxacin, showing the benefits and risks of the use of this compound in veterinary medicine. Animal health and the environmental effects of this stable antibiotic (with half-life as long as 3–9 years in various natural environments) are analyzed, as are the interesting properties of this molecule that are expressed when present in complexes with metals. Recommendations for further research on enrofloxacin are also proposed.

## 1. Introduction

Enrofloxacin is an antibiotic that belongs to the fluoroquinolone group, more specifically the 6-fluoro-7-piperazinyl-4-quinolones [1]. This antibiotic is widely used in veterinary medicine as an antibacterial agent, showing high activity against both Gram-negative and Gram-positive bacteria. This compound is a chemotherapeutic, and it was first synthesized in 1983 from nalidixic acid, while the first enrofloxacin-based medicinal product was marketed in 1991 as an oral drug for poultry by Bayer under the trade name Baytril^®^ [2,3]. Currently, the product is approved by the European Medicines Agency (EMA) in both injectable and tablet forms [4].

Chemically, enrofloxacin is defined as a quinoline monocarboxylic acid that is 1,4-dihydroquinoline-3-carboxylic acid substituted by an *oxo* moiety at position 4, with a fluoro moiety at position 6, a cyclopropyl moiety at position 1, and a 4-ethylpiperazin-1-yl moiety at position 7. Moreover, this antibiotic is a quinoline monocarboxylic acid, a quinolone, an organofluorine compound, an N-alkyl piperazine, and a member of the cyclopropanes [5]. Enrofloxacin is highly lipophilic, and the addition of a carboxylic acid and a tertiary amine contributes to its amphoteric properties [6]. Its crucial chemical and physical properties are shown in Table 1.

## 2. Mechanism of Action

The targets of enrofloxacin, like other quinols, are enzymes that control DNA topology: gyrase and topoisomerase IV. Their activities facilitate the processes of DNA replication, recombination, and gene expression [7]. As heterotetramers, these enzymes are composed of two GyrA and two GyrB subunits, in the case of gyrase, or their homologs ParC and ParE in topoisomerase IV [7,8]. The GyrA and ParC subunits have a tyrosine residue in the active site that is involved in DNA strand breakage, while the GyrB and ParC subunits have the domains required for DNA strand re-ligation. DNA gyrase, by introducing negative supercoils, alleviates topological stresses, allowing replication complexes to move along the DNA. It works by coiling DNA into a positive supercoil and then moving the duplex region accordingly, breaking and rejoining. The speed of the process is regulated by the availability of ATP (the abundance of this nucleotide accelerates the process) [9,10]. Topoisomerase IV activity differs from gyrase activity. Although it can remove positive and negative supercoils, it cannot actively unwind dsDNA. In addition, it has a greater ability to resolve DNA strands [11].

The disruption of enzymatic activities described above is associated with the formation of complexes between DNA and gyrase or topoisomerase IV. When conformational changes occur, quinolone prevents the rejoining of torn DNA strands, and the enzyme itself is trapped on the DNA [12]. In the case of gyrase, rapid inhibition occurs, which is associated with activity upstream of the replication fork. A different situation—the subsequent inhibition of replication—occurs with quinolone–topoisomerase IV-DNA complexes. This is related to the activity of the enzyme downstream of the replication fork. Complex formation is reversible, which is responsible for the bacteriostatic action of the compounds. In contrast, bactericidal activity is considered to be a separate phenomenon from complex formation. The first proposal of the bactericidal effect of enrofloxacin based on free DNA end release, not just complex formation, came from a sedimentation analysis of isolated bacterial nucleoids [13].

Two molecules of enrofloxacin bind non-covalently to the DNA-topoisomerase complex (II or IV) near the tyrosine residue in the active site [7,14]. After binding, enrofloxacin induces conformational changes in the enzyme. This results in the formation of an enrofloxacin–gyrase/topoisomerase IV-DNA complex. The natural consequence of this process is the inhibition of DNA replication [15]. Low concentrations of the antibiotic can trigger the SOS response (a bacteriostatic effect), while concentrations of the antibiotic can fragment the bacterial chromosome, leading to cell death (a bactericidal effect) [16].

In *S. pneumoniae*, topoisomerase IV is the primary target for another quinol, ciprofloxacin, which is, but for variants with a substitution at the C-8 position, the primary target is gyrase [17]. In addition to the structure itself, the difference in the effect of quinolones is due to the fact that gyrases from Gram-positive bacteria are less susceptible to inhibition by quinolones than gyrases from Gram-negative bacteria. Other mechanisms of resistance are worth considering. It was demonstrated that the low level of resistance to quinolones is associated with changes in porins, such as structural changes in OmpF or mutational changes in *marA*, *sox*, and *rob* genes that affect the efflux pump activity [18].

**Figure 1:**
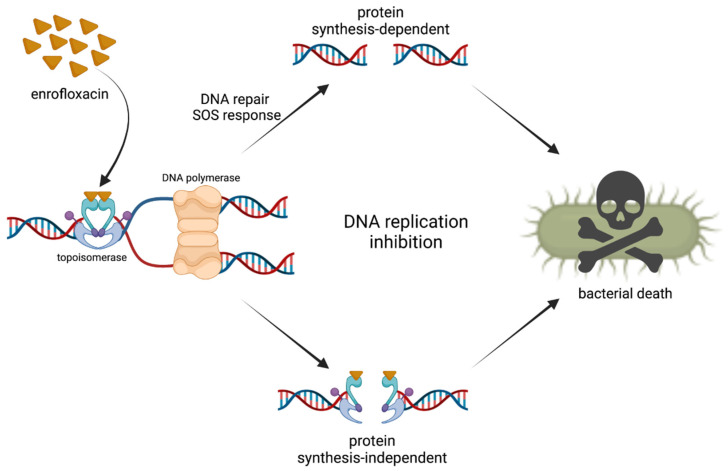
Mechanism of enrofloxacin action. Enrofloxacin binds to the DNA-topoisomerase complex at the cleavage–ligation site. This leads to conformational changes in the enzyme, resulting in the inhibition of DNA replication due to cleavage and the inefficient ligation of DNA. Cell death or DNA repair mechanisms and the S.O.S. response is/are activated depending on concentration. Reproduced according to ref. [19], with modifications.

The mechanism of enrofloxacin action is shown in Figure 1.

## 3. Pharmacokinetics of Enrofloxacin

The pharmacokinetics of enrofloxacin may be influenced by individual factors, such as age, body condition, physiological status, species, drug form, or route of administration [20]. Here, we consider the kinetics of enrofloxacin from the time of drug intake to the time of excretion from the body.

### 3.1. Absorption

Enrofloxacin has high bioavailability and rapid absorption after intramuscular, subcutaneous, and oral administration in most species [21]. However, it has been noted that there are some discrepancies after the oral administration of enrofloxacin in ruminants [20]. Moreover, the bioavailability of the antibiotic may be affected by the nutrition status of the animals, as the bioavailability value in fasted pigs was significantly higher than that in fed pigs [22]. Another factor to consider is the interaction of the antibiotic with ions [23]. Sumano et al. showed a severe decrease in the bioavailability of enrofloxacin in broilers that received the antibiotic dissolved in water with a high content of calcium and magnesium ions [24].

New technologies have been proposed for more efficient drug release and absorption. Prem Kumar et al. used an inert polymer polyvinylpyrrolidone, which, due to its excellent hydrophilic properties, caused a more efficient release of enrofloxacin [25]. On the other hand, Karakurt et al. presented a transdermal sustained release system for enrofloxacin, which is based on a matrix of chitosan and vanillin-crosslinked polyvinyl alcohol [26].

### 3.2. Distribution

The drug efficacy depends on its distribution, especially the spread in its unmetabolized form in the blood and body tissues. It varies depending on the chemical properties of the drug as well as the individual characteristics of the patient [27]. The results of recent analyses that demonstrated the distribution of enrofloxacin in various organs are presented in Table 2. Additionally, enrofloxacin is a concentration-dependent drug and its distribution is affected by protein binding [28]. 

### 3.3. Metabolism

The metabolism of enrofloxacin may vary among species, although it is biotransformed to ciprofloxacin in most animals. Enrofloxacin also has other metabolites, but they are not active [6]. The biotransformation process of enrofloxacin includes N-dealkylation, glucuronide conjugation to the nitrogen in the *para* position of the piperazinyl ring, oxidation in the *ortho* position of the substituted amine, and the opening of the piperazinyl ring [34]. The maximum concentrations of enrofloxacin and its major metabolite ciprofloxacin in enrofloxacin-treated animals are shown in Table 3.

### 3.4. Elimination

The elimination of enrofloxacin varies widely among species. For comparison, the elimination half-life (t_1/2_) after intravenous administration was as follows: in cows, 1.5 h [41], dogs, 2 h [42], sheep, 4.31 h [43], cats, 5.6 h [44], pigs, 9.64 h [45], horses, 9.9 h [46], broilers, 12.84 h [47], African penguins, 13.67 h [48], American alligators, 21.05 h [21], and Atlantic horseshoe crabs, 27.9 h [49].

Moreover, the elimination times of enrofloxacin and ciprofloxacin are different. Poapolathep et al. observed that in green sea turtles, after the intravenous administration of enrofloxacin (7.5 mg/kg), its elimination time was 50.4 h, while the elimination time of ciprofloxacin was 22.6 h. The main reasons for this phenomenon are the different mechanisms of their elimination. The elimination of enrofloxacin is renal, while ciprofloxacin is eliminated by both renal and hepatic pathways. However, Trouchon and Lefebvre reported that both enrofloxacin and ciprofloxacin undergo intestinal recirculation via bile excretion [3]. In their recent report, Yang et al. showed that in Yellow River carp, bile excretion might be the primary elimination route of enrofloxacin [33].

## 4. Efficacy of Enrofloxacin in Veterinary Medicine

**Figure 2:**
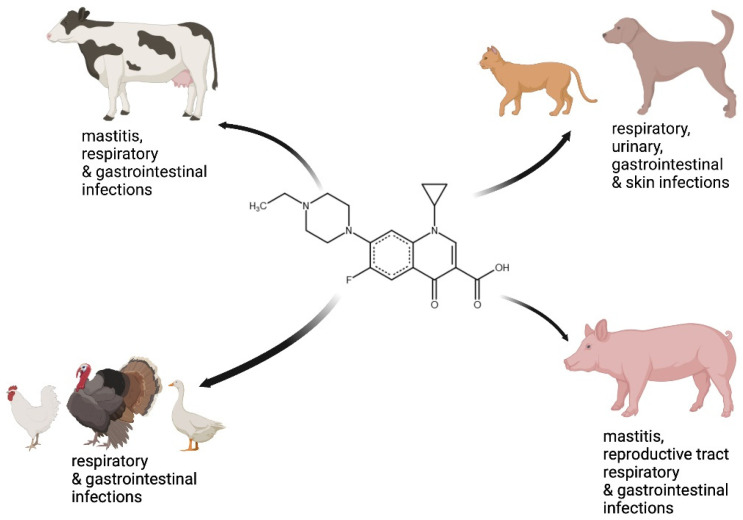
Diseases of animals that can be treated with enrofloxacin.

Enrofloxacin is an antibacterial agent with a broad spectrum of activity covering many Gram-negative and Gram-positive bacterial species. This antibiotic is commonly used to treat dogs, cats, cows and calves, domestic chickens and turkeys, and exotic animals such as parrots, alligators, and snakes, but it is not approved for use in humans [21,50,51,52,53,54,55,56]. The latter decision is due to the toxic effects of enrofloxacin in humans, especially neurotoxicity, genotoxicity, and building up in the cartilage, as well as because of low solubility and low bioavailability [25,57]. Therefore, it is unlikely that this compound might be used to treat patients, as the risk of severe adverse effects would be too high, especially as relatively large doses would have to be employed. Nevertheless, other fluoroquinolones, including an enrofloxacin metabolite called ciprofloxacin, have been approved as drugs for humans, as they appear to be less toxic and possess more favorable pharmacological parameters. The target sites for the treatment of the bacterial infections of animals with enrofloxacin are shown in Figure 2.

The minimum inhibitory concentration (MIC) value for various bacteria is relatively low. *Escherichia coli* strains isolated from the bovine uterus showed MIC values ≤ 0.25 µg/mL, which was analogous to the results obtained by Liu et al. for *E. coli* strains causing thigh infections in mice [58,59]. For *Salmonella* strains, the MIC value varied between 0.06 and 0.25 µg/mL [60,61]. Significantly higher MIC values were obtained for *Pseudomonas aeruginosa* strains, ranging between 1 and 4 mg/mL [60,62].

Enrofloxacin is highly effective in the treatment of respiratory, urinary, gastrointestinal, and skin inflammation in dogs and cats [3]. Westropp et al. tested the potential of enrofloxacin against urinary tract infections caused by aerobic bacteria in dogs. Infected animals (10^3^ CFU/mL) received the antibiotic *per os* at a dose of 18–20 mg/kg for 3 days. Of all infected animals treated with enrofloxacin, up to 88.6% of individuals recovered [63]. One recurrent skin disease in dogs is pyoderma, caused by *Staphylococcus intermedius*. It has been demonstrated that the use of enrofloxacin at a dose of 5 mg/kg for a minimum of one week can be an effective treatment for this disease [64]. Conjunctivitis in cats, caused by *Chlamydophila felis*, can also be effectively treated with enrofloxacin. This was demonstrated by Gerhardt et al. who, using a dose of 5 mg/kg, observed that 92% of affected cats recovered [65].

In cattle, enrofloxacin has been used to control bacterial infections of the mastitis, respiratory, and gastrointestinal tracts [34]. *Mannheimia haemolytica* is the bacterium most commonly isolated from cattle suffering from bovine respiratory disease (BRD). It was shown that the subcutaneous application of enrofloxacin (at 7.5 mg/kg) is effective against this bacterial pathogen [52]. Initial attempts to treat mastitis with enrofloxacin were unsuccessful [66,67]. However, a recent study by Alfonseca-Silva et al. confirmed the effectiveness of this antibiotic in the treatment of mastitis, although the authors used a slightly different approach. The antibiotic (in the form of hydrochloride dihydrate) was administered twice daily for 5 days via intramammary infusions (infusions into the udder) at a dose of 300 mg. The authors showed that 65% of cattle treated with enrofloxacin recovered, while the additional introduction of ceftiofur-HCl led to a 100% recovery [68].

The effect of enrofloxacin has also been confirmed in bacterial diseases of poultry, such as salmonellosis, colibacteriosis, and pastelosis [3]. Marien et al. administered this antibiotic to animals in drinking water at a dose of 10 mg/kg for 3 or 5 days. The authors confirmed the efficacy of enrofloxacin in treating turkeys triple infected with *E. coli*, *Ornithobacterium rhinotracheale*, and avian pneumovirus (APV). Moreover, they demonstrated that enrofloxacin was a more effective bactericide than florfenicol (at 20 mg/kg per 5 days) or amoxicillin (at 20 mg/kg per 5 days) [69]. Similar results for the efficacy of enrofloxacin in the eradication of *O. rhinotracheale* were obtained by Garmyn et al. [70]. Enrofloxacin is particularly effective against *Salmonella* strains. Li et al. demonstrated that a seven-day administration of the antibiotic at a dose of 100 mg/kg not only effectively treated salmonellosis, but additionally minimized the possibility of resistance development in *S.* Typhimurium [71].

Enrofloxacin has also been used in controlling bacterial infections in exotic animals [34]. Asian house geckos are endangered by *Enterococcus lacertideformus*, a pathogen that causes a systemic infection that is usually fatal. Agius et al. treated infected animals with enrofloxacin per os, once daily at a dose of 10 mg/kg for 21 days. The authors showed that the use of this antibiotic was effective in 83.6% of geckos, while the effectiveness of treatment with amoxicillin–clavulanic acid was 58.2%, rifampicin 45.5%, and clarithromycin 26.5% [72]. This antibiotic has also been used to treat lung disease in sea turtles and Indian pythons [73,74].

It is important to note that enrofloxacin should only be used in justified situations. Particularly, it can be employed to treat infections resistant to other antibacterial agents after testing the susceptibility of infecting bacterial strains. The caution is necessary as this antibiotic may affect the gut microbiome as well as increase the risk of developing resistance by bacteria. Morales-Barrera et al. showed that the prophylactic use of enrofloxacin in turkeys at a dose of 50 mg/kg increased the susceptibility of animals to *Salmonella* infections [75]. A recent study by Janssen et al. indicated that the use of enrofloxacin may affect the development of the resistance of commensal *E. coli* strains in pigs [76]. Analogous results were obtained by Lin et al., who noted an increase in the number of ciprofloxacin-resistant *E. coli* strains in pigs treated with enrofloxacin [77], and Kaspersen et al., who found that even a single treatment with enrofloxacin significantly increased the probability of developing quinolone resistance in commensal *E. coli* strains [78]. Pomorska-Mól et al. also observed that the use of enrofloxacin at the time of vaccination for Aujeszky’s disease significantly delayed the humoral response and decreased the level of IFN-γ in pigs [79].

## 5. Mechanisms of Resistance to Enrofloxacin

The ability of bacteria to develop resistance to antibiotics, including fluoroquinolones, is becoming a global public health problem across the world. The main causes of this phenomenon are the inappropriate incorporation of antibiotics in treatment, as well as the misuse of huge amounts of antibiotics for preventive purposes in animal breeding [80]. The use of enrofloxacin in veterinary treatment is quite controversial. The increase in enrofloxacin-resistant strains led to the withdrawal of the use of this antibiotic in the United States in 2005 [81], while its use in food-producing animals was never allowed in Australia [82,83]. Nevertheless, the presence of strains resistant to fluoroquinolones has been reported in the following years in these countries [84]. Enrofloxacin is accepted by the European Medicines Agency (EMA) for veterinary use in injectable and tablet forms. However, on 14 February 2018, the Agency’s Committee for Veterinary Medicinal Products (CVMP) stated that medicinal products containing enrofloxacin should no longer be used in chickens and turkeys for the treatment of *E. coli* infections [85].

In bacteria, the development of resistance mechanisms to fluoroquinolones is caused by (i) the presence of resistance genes (*qnr*) in plasmids, protecting the target topoisomerase, (ii) the presence of efflux pump systems encoded by plasmids, (iii) the presence of a gene (*aac(6′)-Ib-cr*) encoding an enzyme that modifies fluoroquinolones, and (iv) the appearance of mutations in the quinolone resistance determinant region (QRDR) within the subunits forming topoisomerases II and IV [86,87,88,89,90,91]. These mechanisms can be either chromosome- or plasmid-borne [88].

The plasmid-borne fluoroquinolone resistance gene (*qnr*) codes for a 219-aa protein that protects both DNA gyrase and topoisomerase IV from the antibiotic by destabilizing the gyrase–antibiotic complex [92]. Three major plasmid gene families are known to encode the proteins responsible for fluoroquinolone resistance: *qnrA*, *qnrB*, and *qnrS* [93]. The presence of these genes was identified in enrofloxacin-resistant *E. coli* strains [94,95,96].

The efflux pump system decreases the intracellular concentration of fluoroquinolones by transporting the antibiotic from the cell to the environment. The mechanisms of resistance to enrofloxacin associated with the increased expression of efflux pumps have been identified in *S.* Typhimurium [97], *S.* Entiritidis [98], *E. coli* [99,100], and *Enterococcus* strains [101].

The aminoglycoside acetyltransferase AAC(6′)-Ib-cr is an enzyme capable of N-acetylating piperazine-substituted quinolones, leading to reduced bacterial sensitivity to antibiotics, including enrofloxacin [102]. AAC(6′)-Ib-cr has two amino acid changes, Trp102Arg and Asp179Tyr, which together are necessary for the enzyme’s ability to acetylate the antibiotic [103]. The presence of a gene encoding AAC(6′)-Ib-cr was confirmed in clinical enrofloxacin-resistant strains of *E. coli* [104] and *S.* Indiana [105].

The genes encoding the topoisomerase II (*gyrA*) and IV (*parC*) subunits contain specific domains called quinolone resistance determinant regions (QRDRs). Resistance to quinolones arises due to amino acid substitutions within these sequences, which leads to the abnormal conformation of the subunits and the reduced binding affinity of the drug to the DNA-gyrase or DNA-topoisomerase IV complex [15,89]. The presence of such mutations was demonstrated in enrofloxacin-resistant strains of *Pseudomonas aeruginosa* isolated from companion dogs [106]. Mutations within the *gyrB* and *parE* genes can also cause resistance to quinolones, but they occur less frequently than mutations within *gyrA* and *parC* [15].

It is important to stress that resistance to one fluoroquinolone (like enrofloxacin) usually means the insensitivity of bacterial cells to other antibiotics from this group. This phenomenon is due to the structural identity of the fluoroquinolone backbone present in all these compounds, which determines the mechanisms of the resistance developed by bacteria. Thus, products of the *qnr* genes, a family of the pentapeptide repeat proteins, can effectively protect DNA gyrase and topoisomerase IV, targets of fluoroquinolones, against any compounds from this group [93]. Similarly, specific efflux pumps can remove all fluoroquinolones from bacterial cells. The *aac(6′)-Ib-cr* gene product is an enzyme capable of modifying the fluoroquinolone backbone, irrespective of the side moieties present in different antibiotics belonging to this group. Finally, since all fluoroquinolones interact with the same region of topoisomerases (called QRDR), mutations causing changes in the tertiary structures of this region of the enzymes prevent the binding of any fluoroquinolone-like molecules. Many examples confirm this statement. It has been observed that selection pressure caused by the use of enrofloxacin has led to resistance to many of the fluoroquinolones used in veterinary and medicine [107,108]. It has been reported that *Campylobacter* strains resistant to enrofloxacin were also resistant to ciprofloxacin and other fluoroquinolones used in medicine, as isolates from enrofloxacin-treated hens developed the p.Asp90Asn mutation in the *gyrA* gene, leading to the development of bacterial resistance to other fluoroquinolones [109]. Moreover, *Salmonella* strains treated with enrofloxacin developed resistance to nalidixic acid and ciprofloxacin [110]. In fact, the incidences of quinolone-resistant *Salmonella* increased significantly after the approval of the use of quinolones in livestock [110]. Importantly, it was suggested that the use of enrofloxacin in the food of animals affects the occurrence of ciprofloxacin resistance in zoonotic *Salmonella*, resulting in human infections [111].

Irrespective of the mechanisms of resistance to enrofloxacin, the appearance of resistant bacteria should be considered to be a potentially dangerous phenomenon. Since enrofloxacin is considered to be a strong antibacterial compound, it might be potentially used under conditions when other antibiotics fail to eliminate pathogenic bacteria [3]. However, the overuse of this drug can likely lead to the selection and spread of antibiotic-resistant bacteria, which could potentially lead to a decrease in its general efficacy as a treatment for animals. The appearance of enrofloxacin-resistant bacterial mutants and their spread has been reported after the use of this antibiotic for the treatment of chicken [108] or ducks [112]. Especially problematic is the development of resistance under sub-inhibitory concentrations of enrofloxacin [98], as such conditions may occur in places with its therapeutic use. This is particularly dangerous, since bacteria resistant to enrofloxacin are also resistant to other fluoroquinolones [98]. Therefore, despite the fact that enrofloxacin has been approved for use only in veterinary medicine, not in humans [34], the spread of resistant bacteria is problematic as other fluoroquinolones, like ciprofloxacin, are antibiotics employed in the treatment of humans. In this light, one might predict that virulent bacterial strains which gained resistance when present in animals might transfer to humans, causing hard-to-treat infections. Moreover, since fluoroquinolone-resistance mechanisms can be plasmid-borne [88,89,90,91,92,93,94,95,96,97,98,99,100,101], this feature can be easily transferred in the environment to other bacterial strains or even species, possibly causing the selection of dangerous human pathogens insensitive to a large group of antibiotics.

## 6. The Safety of Enrofloxacin Use

There is no doubt that enrofloxacin is an effective antibacterial agent. However, organisms treated with this antibiotic are prone to many adverse effects. The most common adverse effects of enrofloxacin include changes in the skeletal, reproductive, gastrointestinal, immune, and nervous systems [3]. However, it should be kept in mind that the occurrence of adverse effects is mainly dependent on the dose used and the frequency of antibiotic administration. In this chapter, we will discuss the known adverse effects of enrofloxacin in farm animals and in the environment.

### 6.1. Adverse Effects in Farm Animals

#### 6.1.1. Skeletal System

One of the most commonly confirmed adverse effects of enrofloxacin are changes in the skeletal system. The confirmed in vitro and in vivo changes mainly concern arthropathy, tendon damage, and the destruction and degeneration of articular cartilage [3].

Among the most common enrofloxacin-induced degenerations are articular cartilage lesions in young animals. Histopathological analyses have confirmed that this antibiotic causes a decrease in matrix proteoglycans, total cartilage thickness, a decrease in the number of chondrocytes, the appearance of spindle cells, and an increase in the number of gaps and voids in the articular cartilage matrix in young lambs [113]. Moreover, safety studies of enrofloxacin confirmed that the use of this antibiotic causes a drastic decrease in the viability of chondrocytes, induces their apoptosis and DNA fragmentation, causes an increase in pro-inflammatory factors, such as Il-1β, TNF, and MMP3, and can affect the shape of the actin and vimentin cytoskeleton [114,115,116,117]. Analogous results were obtained in safety studies of enrofloxacin in growing hens. It was shown that the use of this antibiotic led to morphological changes in chondrocytes within the cytoplasm and cell nucleus, as well as a loss of proteoglycan [118].

Enrofloxacin is also an agent that causes pathological changes in tendon cells. It has been shown that the use of the antibiotic in horses inhibits cell proliferation and also induces morphological changes, including cell membrane perforation [119]

It is also worth noting that enrofloxacin negatively affects bone marrow cells. Han and Joo demonstrated that the use of enrofloxacin causes a decrease in metabolic activity and stimulates the death of mouse bone marrow cells, as well as decreasing the number of CD11b + Gr1+ neutrophils [120].

Despite such severe adverse effects, the potential of enrofloxacin has been successfully exploited in canine osteosarcoma studies. Won Seo et al. demonstrated that the antibiotic induces apoptosis and inhibits *p21*(WAF1) expression in osteosarcoma cells, which resulted in decreased cell proliferation [121].

#### 6.1.2. Reproductive System

Enrofloxacin shows deleterious effects mainly on the male reproductive system; however, the exact mechanism of this process is not known [122,123,124]. Aral et al. performed one of the first studies on the effects of enrofloxacin on semen quality in mice. The authors showed that the application of this antibiotic at a dose of 150 mg/kg caused a decrease in motility and sperm count, as well as an increase in the number of abnormal sperm. Moreover, histopathological examination confirmed the degeneration of seminal tubules, incomplete spermatogenesis, and a decreased sperm concentration in seminal tubules [123]. Analogous results were obtained by Rugsung et al. in a rat model, further demonstrating that enrofloxacin is able to decrease testosterone levels [124].

However, it is important to note that enrofloxacin can also affect the cells of the female reproductive system. Tkachenko et al. demonstrated that the use of the antibiotic adversely affects oocytes in the common marmoset. They observed both a decrease in the number of oocytes and morphological changes, including the abnormal structure of the meiotic spindle [125]. Additionally, it was observed that enrofloxacin induced cytotoxicity in bovine cumulus cells, as evidenced by changes in mitochondrial activity [126].

Enrofloxacin may also adversely affect avian embryonic development. The cardiotoxic effect of the antibiotic on chicks was demonstrated. The accelerated heart rate of embryos resulted in chicks hatching too fast, and thus their drastically low survival rate. Moreover, some of the chicks showed joint deformities, as well as abnormal blood biochemical parameters, such as hyperproteinemia, mild hyperglycemia, and increased blood urea nitrogen/uric acid ratio [127].

In specific cases, enrofloxacin can be used to treat infections in pregnant animals. The short-term administration of enrofloxacin to late pregnant mares (at 260 days of gestation) has been shown to cause placental penetration of enrofloxacin; however, it was not toxic to the fetus [128].

Extreme caution should be exercised when using enrofloxacin during the reproductive period. As outlined above, enrofloxacin may affect not only the quality of germ cells and the normal anatomy of the reproductive system, but also fetal development.

#### 6.1.3. Retinopathies

Enrofloxacin is one of the agents that causes retinopathy and blindness in domestic cats. The characteristic pathological change associated with the toxic effects of the antibiotic is a reduction of the outer nuclear membrane of the retina [3,129]. Moreover, histopathological studies have shown that the antibiotic leads to the loss of photoreceptors, as well as the hypertrophy and proliferation of the retinal pigment epithelium. Sometimes, a return of vision is observed; however, retinal degeneration persists or progresses [130,131]. Interestingly, retinopathic changes are not seen in non-domestic cats. Newkirk et al. analyzed the effect of enrofloxacin on the thickness and morphology of the retinal layer in lions and panthers. They showed that the antibiotic had no toxic effects on the retina and could be safely used in these animals [129].

#### 6.1.4. Hepatotoxicity

The liver is the main organ responsible for detoxification and is, therefore, exposed to many adverse substances. Enrofloxacin at high concentrations can accumulate in the liver, leading to a number of hepatotoxic changes, and can also alter liver enzyme activity [132]. Studies on grass carp liver cells have shown that this antibiotic is able to induce hepatocyte apoptosis through a mitochondrial-dependent pathway, as well as cause a drastic change in the values of biochemical parameters, such as an increase in the levels of lactate dehydrogenase (LDH) and malondialdehyde (MDA), a decrease in the total mitochondrial membrane potential (DJm), the generation of reactive oxygen species (ROS) (at a dose of 200 µg/mL), and a decrease in the total antioxidant capacity (T-AOC) [132]. Enrofloxacin may also interfere with the activity of cytochrome P450 enzymes, which are responsible for drug metabolism [133,134]. This antibiotic has been shown to be able to inhibit CYP1A2 in rats [135], CYP3A in sea bass and hens [136,137], and also CYP3A4 and CYP2E1 in pigs [134]. Furthermore, it is capable of upregulating microsomal NADPH-cytochrome C reductase (NCCR) and inhibiting microsomal erythromycin N-demethylase (ERND) and aminopyrine N-demethylase (AND) activities. Additionally, a proteomic analysis revealed the increased expression of carboxylesterase (CES) and alpha-enolase (ENO1) proteins as a response to enrofloxacin-induced stress [138].

#### 6.1.5. Immune System

The use of enrofloxacin can also induce an inflammatory response at the injection site and affect the cell count and protein levels of the immune system. Enrofloxacin has both cytotoxic and genotoxic effects on the lymphocyte population. It has been shown to affect the formation of chromosomal aberrations, mainly chromatid and chromosome breaks, as well as chromosomal gaps in human lymphocytes [139]. Moreover, the in vitro effects of enrofloxacin-induced bovine lymphocyte death and DNA damage, regardless of the concentration used, have been reported [126]. The exposure of common carp macrophages to enrofloxacin resulted in the activation of the NF-κB pathway and the induction of an NF-κB-based immune response that included reactive oxygen species formation and cytokine expression [140].

An inflammatory response at the site of antibiotic injection has been confirmed in pigs [3]. Recent studies indicated that this effect could also occur in fish. The intramuscular injection of enrofloxacin in striped bass led to hemorrhage, necrosis, and inflammation [141].

### 6.2. Other Adverse Effects in Veterinary Medicine

The list of adverse effects caused by enrofloxacin is vast. It is important to note that enrofloxacin is also able to affect the nervous system, heart function, vitamin levels in the body, and behavior. The induction of epileptic seizures and interaction with serum albumin were also noted. A list of studies in which adverse effects caused by the use of enrofloxacin have been observed is presented in Table 4.

### 6.3. Environmental Adverse Effects

Enrofloxacin, like many antibiotics, is considered to be an environmental toxin. Due to the overuse and misuse of antimicrobial compounds, elevated levels of this antibiotic have been increasingly observed in various environments, such as soils, groundwater, sewage treatment plants, and farms [150]. The half-life of enrofloxacin, which ranges between 1155 and 3466 days (depending on environmental conditions), appears to be extremely dangerous [3,151]. One of the main sources of enrofloxacin contamination is poultry litter, which is widely used as an agricultural fertilizer due to its high content of micro- and macro-elements and its ability to improve soil pH [152]. The negative effect of enrofloxacin on the population of soil and aquaculture organisms has been confirmed.

It has been shown that the enrofloxacin present in poultry litter has a toxic effect on earthworms (*Eisenia andrei*), causing deterrence, biomass loss, and death depending on the dose applied [153]. Similar results were found by Gao et al., who studied the effect of enrofloxacin on growth rate and catalase activity in *Eisenia fetida*. The authors further confirmed that earthworm intestinal tissues are more sensitive to enrofloxacin than body wall muscle tissues [154].

The continued entry of enrofloxacin into the aquatic environment poses a long-term threat to the organisms living there. The removal of enrofloxacin and other fluoroquinolone antibiotics is a very complex process, and it is not always effective [155,156]. Therefore, the biocontrol of organisms exposed to these antibiotics is crucial. The effects of enrofloxacin on the growth of giant freshwater shrimp were investigated. The analysis confirmed that enrofloxacin at the high doses to which the shrimp were exposed caused stunted animal growth, gill and liver damage, and the induction of hepatopancreatic cell apoptosis. Moreover, the induction of oxidative stress by enrofloxacin is believed to be a likely cause of this phenomenon [157]. Interestingly, a safety analysis of this antibiotic on anuran amphibian larvae showed that the environmental concentration of enrofloxacin (10 µg/L) affected the development, size, shape, and growth of larvae, as well as inhibiting the activity of antioxidant enzymes [155]. It was also proposed that in *Daphnia magna*, enrofloxacin can cause high reproductive toxicity, as well as genetic or epigenetic changes that will only produce effects in subsequent generations [158,159,160].

The increased concentrations of enrofloxacin in aquatic environments pose serious health risks to humans and livestock. Although enrofloxacin adversely affects aquacultures, data on its concentration in various bodies of water are limited. The concentrations of this antibiotic in several aquatic environments have been investigated, and it appears that the main sites of accumulation of enrofloxacin are surface waters, such as rivers, where the levels range between 12 ng/L and 4.24 µg/L [161], creeks where the concentrations are 17–216 ng/L [162], and ponds where the concentration is about 0.50 µg/L [163]. The appearance of enrofloxacin in tap water at concentrations ranging from 2.0 to 4.0 ng/L has also been reported [164]. However, the largest reservoirs of enrofloxacin are domestic wastewaters, municipal wastewaters, as well as hospital wastewaters, where enrofloxacin can occur at concentrations as high as 100 µg/L [165].

## 7. Interactions with Metal Ions

Both antibiotics and heavy metals exhibit long-term cytotoxicity to the environment. Therefore, potential interactions between antibiotics and heavy metals are being increasingly investigated. Enrofloxacin is one of the antibiotics that can interact with metal ions. The most relevant aspects of this phenomenon to be discussed are the effects of the antibiotic–metal mixture on microbial populations, the importance of the degradation process of enrofloxacin in combination with metals, and the application of this phenomenon in laboratory diagnostics.

### 7.1. Effects on Microbial Populations

The use of enrofloxacin in combination with metals may affect the survival of microorganisms. Wei et al. conducted a study in which they examined the toxicity of enrofloxacin, copper (Cu), and the enrofloxacin-Cu combination. The authors showed that the toxicity of the antibiotic–metal combination on the soil bacterial population was higher than that of the antibiotic alone, indicating a synergistic effect of the antibiotic–metal complex. However, in fungal populations, it has been observed that a combination of enrofloxacin and metal has a predominantly antagonistic effect [150]. A similar study was performed by Wang et al., who tested the toxicity of enrofloxacin in combination with cadmium. The authors showed that the interaction between the antibiotic and the metal was antagonistic and that the combined contamination of soil with cadmium and enrofloxacin reduced their toxic effects on soil microorganisms [166].

A recent study by Yan et al. showed the promising therapeutic potential of enrofloxacin–calcium complex [Ca(EFX)_2_(H_2_O)_4_]. This compound was tested on *E. coli* and *S.* Typhi strains. It is noteworthy that the use of an enrofloxacin complex with calcium in a rat model resulted in a reduction in its acute toxicity and the rate of binding to plasma, as well as better distribution of the drug. Moreover, this complex showed greater efficacy against *E. coli* in the chicken model than enrofloxacin alone (cure rates were 88% and 78%, respectively) [167].

The following facts have also been confirmed: the interaction of enrofloxacin with Cu^2+^ ions via pyridone and carboxylate moieties [168], the modulation of binding of this antibiotic to the outer membrane protein OmpF of *E. coli* in the presence of Mg^2+^ ions [169], and the increased adsorption of enrofloxacin in calcareous soil in the presence of Zn(II) [170] have all been observed. The list of other studies reporting the significant effects of enrofloxacin in combination with metals is presented in Table 5.

### 7.2. Use of Metal–Enrofloxacin Interactions in Laboratory Diagnostics

The ability of enrofloxacin to interact with metals has also been used in applications for laboratory diagnostics. Tong et al. proposed a synchronous fluorescence method for the determination of enrofloxacin concentrations which is based on yttrium-induced luminescence. When yttrium in the form of Y^3+^ ion is added to an enrofloxacin solution, the fluorescence of the antibiotic is significantly enhanced. This method allows one to determine the levels of enrofloxacin in pharmaceutical preparations, but also allows the evaluation of the level of this antibiotic in milk [176]. Rezaei and Mokharti also proposed a method for the determination of enrofloxacin using a flow-injection system. It is based on the rapid reduction of Ru(phen)_3_^3+^, which is formed in the reaction between Ru(phen)_3_^2+^ and acidic Ce(IV) by enrofloxacin, leading to strong chemiluminescence. The method has been used to evaluate enrofloxacin levels in plasma and poultry meat [177].

### 7.3. Exploitation of Metal–Enrofloxacin Interaction in Antibiotic Degradation

The overuse of antibiotics in veterinary medicine has resulted in the appearance of these drugs in the environment. The main reason for this phenomenon is the use of contaminated animal waste as natural fertilizers. Moreover, these drugs enter the environment through effluents from wastewater treatment plants that are unable to handle such contaminants [178,179]. Enrofloxacin, like most fluoroquinolones, is considered to be an environmental contaminant of increasing importance. Its presence has been found in wastewater, agricultural soils, and animal manure [151,178,180]. Due to the potential environmental toxicity of enrofloxacin, alternative methods are being sought to degrade this antibiotic. Previous attempts have included biodegradation processes and physical degradation using metals.

Alexandrino et al. used a population of soil microorganisms obtained from the rhizosphere sludge of plants from experimentally constructed wetlands that were designed to treat farm wastewater to biodegrade enrofloxacin. Metagenomic analysis identified the major taxonomic groups that are associated with enrofloxacin degradation. Despite the confirmation of the biodegradation of enrofloxacin, the efficiency of this process was low, at the level of about 40–55% [178].

However, considerably higher efficiencies of enrofloxacin degradation were obtained with physical methods using metals. One way to remove enrofloxacin from the environment is photodegradation. This process leads to the degradation of molecules when exposed to light. Sturini et al. examined the kinetics of the photodegradation process of enrofloxacin in untreated river water under sunlight and under the same conditions in the presence of titanium oxide (TiO_2_). The authors proved that the use of titanium oxide accelerated the photocatalysis process two fold [166]. Similar results were obtained by Yu et al., who confirmed the role of titanium oxide, in the form of an Fe_3_O_4_@TiO_2_-GO (FTG) catalyst, in the photocatalysis of enrofloxacin [181].

Yang et al. confirmed the ability of Fe(VI) to oxidize enrofloxacin, leading to its complete degradation. Moreover, the resulting oxidation products of the antibiotic have no antibacterial properties, and its toxicity in source waters was removed [182]. On the other hand, Scisenko et al. investigated the role of Fe(III) on the photolysis rate of enrofloxacin. Interestingly, the half-life of the antibiotic bound to Fe(III) increased from about 22 min to 2.1 h. Only the addition of H_2_O_2_ resulted in a more efficient degradation of the antibiotic–metal complex [183].

### 7.4. Translational Implications of Metal–Enrofloxacin Interactions

The examples described above demonstrated how important it is to study the interactions between metals and antibiotics. The action of a metal–enrofloxacin complex, or a metal–enrofloxacin interaction, can not only affect the degradation time of an antibiotic, but also alter its toxicity and antibacterial properties. Importantly, these properties can be either enhanced or impaired, depending on the kind of microorganisms and the nature of the metals.

The interactions of enrofloxacin with metal ions may affect not only the activity, but also the bioavailability of this antibiotic. It has been observed that hard water, in which the concentrations of Mg^2+^ and Ca^2+^ ions are extremely high, causes the formation of complexes composed of the antibiotic with these ions. Although the formation of such a complex compound did not affect the antibacterial activity of the antibiotic, it drastically reduced its absorption by the epithelium of the gastrointestinal tract and lowered its maximum concentration in plasma [24]. Such a phenomenon might lead to a decrease in the effectiveness of the therapy, and could also increase the possibility of developing microbial resistance to the drug. Therefore, when using enrofloxacin in animal breeding, it is necessary to supply water of appropriate quality.

## 8. Concluding Remarks and Future Perspective

Enrofloxacin is undoubtedly an effective antibacterial agent, as evidenced by a huge number of in vivo studies. However, there is often more harm than benefit in using this antibiotic. When using enrofloxacin, there are many factors to consider that may influence both the success of the treatment and the occurrence of potential adverse effects [184].

Consideration should be given to whether the use of this antibiotic as a first-choice drug is warranted. The use of enrofloxacin is certainly effective due to its broad spectrum of action, as well as its fairly favorable pharmacokinetics. However, its use can be very taxing on the body and can cause a tremendous number of adverse effects, including skeletal, reproductive, immune, and digestive changes. Additionally, the use of enrofloxacin may increase the likelihood of resistance development in commensal bacteria. It is important to note that enrofloxacin may interact with other drugs and metals, which may affect the success of the therapy. Another aspect that should be considered is the high environmental toxicity of enrofloxacin. This antibiotic has a long half-life and also adversely affects the biocenosis of marine and terrestrial ecosystems.

Future research on enrofloxacin should include a search for new strategies that will reduce the toxicity of this antibiotic, as well as allow its safer and more efficient degradation. Molecular mechanisms of enrofloxacin-mediated toxic effects on animal and human cells should be determined to make such a search effective. Possible targeted chemical modifications of this effective antimicrobial agent might enhance its efficacy, restrict the possibilities of the development of bacterial resistance, and increase its safety parameters by reducing adverse effects.

On one hand, enrofloxacin is a strong-acting antibacterial agent, which is its advantage, but on the other hand, it is also more toxic to eukaryotic cells than other fluoroquinolones (and many other groups of antibiotics), causing severe adverse effects in animals and revealing high toxicity to humans. The latter feature, combined with its low solubility and bioavailability, precludes its use to treat humans where other similar molecules, including enrofloxacin’s metabolite, ciprofloxacin, are definitely a better choice at the moment. However, some features of enrofloxacin, especially its efficiency in killing various groups of bacteria, both Gram positive and Gram negative, tempts us to propose that further works should be dedicated to chemical modifications of this molecule to minimize its toxicity to eukaryotic cells while retaining the strong antibacterial activity, which might lead to the development of novel, promising therapeutic agent(s).

## Figures and Tables

**Table 1:** Selected physicochemical properties of enrofloxacin (data based on ref. [5]).

Property	Value
Molecular formula	C_19_H_22_FN_3_O_3_
Molecular weight	359.3 g/mol
Chemical safety	Irritant, health hazard, environmental hazard
Color/form	Pale yellow crystals
Melting point	220 °C

**Table 2:** Penetration of enrofloxacin into various organs of animals (nd—not determined).

Confirmed Presence of Enrofloxacin in Organs	Volume of Distribution (V_d_) (L/kg)	Animal Model	Reference
Serum, liver, kidney, lung, brain, breast muscle, thigh muscle, spleen, and heart	5.07	Broiler chicken	[29]
Plasma, muscle, skin, and liver	2.21	Largemouth bass	[30]
Plasma, hepatopancreas, muscle, gill, and ovary	nd	Ridgetail white prawn	[31]
Plasma, skin, muscle, liver, kidney, and gut	nd	Rainbow trout	[32]
Plasma, skin, muscle, gill, kidney, liver, bile, and gut	nd	Yellow river carp	[33]

**Table 3:** Maximum concentrations (C_max_) of enrofloxacin and ciprofloxacin in enrofloxacin-treated animal models.

Applied Dose of Enrofloxacin (mg/kg)	C_max_ Ciprofloxacin (μg/mL)	C_max_ Enrofloxacin (μg/mL)	Administration Method	Animal Model	Reference
7.5	0.36	2.59	Intramuscular	Green sea turtles	[35]
5	no data	2.33	Intramuscular	Freshwater crocodiles	[36]
10	0.24	12.31	*per os*	Asian house geckos	[37]
10	<0.1	67.90	Subcutaneous	Eastern box turtles	[38]
20	2.28	5.36	Subcutaneous	Prairie dogs	[39]
10	<0.1	90.92	Intracelomic	Green sea urchin	[40]

**Table 4:** Studies in which adverse effects caused by the use of enrofloxacin were observed.

Study Type	Animal Model	Observed Effect	Reference
in vitro	Cattle	Toxic interaction with serum albumin	[142]
	Cattle	Cytotoxicity on embryonic limb bud cells and midbrain cells	[143]
in vivo	Rats	Slight decrease in liver vitamin A and E levels	[144]
	Elephants	Anorexia, decreased water intake, constipation, depression, ataxia, limb paresis, and tremors	[145]
	Genetic Absence Epilepsy Rats from Strasbourg (GAERS)	Induction of clonic seizures	[146]
	Dogs	Alteration of cardiac ventricular depolarization and repolarization, as well as increasing the risk of ventricular arrhythmias.	[147]
	*Acipenser baerii*	Structural damage to liver, kidney, and cartilage	[148]
	*Danio rerio*	Changes in the catalytic activity of glutathione peroxidase and glutathione S-transferase	[149]

**Table 5:** Examples of studies in which a significant effect of metal–enrofloxacin interaction was observed.

Metal	Observed Effects	Reference
Co(II) and Ni(II)	(1) Broader spectrum of antibacterial and antifungal activity against: *E. coli*, *S. aureus*, *P. aeruginosa,* and *C. albicans* (2) No cytotoxic effect of tested complexes on L929 cell line	[171]
Cu(II)	(1) Increased antibacterial activity against *E. coli* and *Salmonella* (2) Enhanced cytotoxic potential against breast cancer cell line (MCF-7)	[172]
Cd	(1) Increased bioaccumulation of Cd caused by enrofloxacin in earthworms (2) Enhancement of oxidative stress induced by Cd	[173]
Cd	(1) Increased cytotoxicity of the complex compared to the antibiotic alone (2) Most of the interactions observed were antagonistic reactions	[174]
Cu	(1) Application of the complex increased toxicity to soil enzymes	[175]

## Data Availability

Not applicable.
